# Impact of the clinical context on the 14-3-3 test for the diagnosis of sporadic CJD

**DOI:** 10.1186/1471-2377-6-25

**Published:** 2006-07-26

**Authors:** Natividad Cuadrado-Corrales, Adolfo Jiménez-Huete, Carmen Albo, Rafael Hortigüela, Luz Vega, Laura Cerrato, Maríajosé Sierra-Moros, Alberto Rábano, Jesús de Pedro-Cuesta, Miguel Calero

**Affiliations:** 1 Centro Nacional de Microbiología, Instituto de Salud Carlos III, Majadahonda, Madrid, Spain; 2 Servicio de Neurología, Hospital Ruber Internacional, Madrid, Spain; 3 Dirección General de Salud Pública. Ministerio de Sanidad y Consumo, Madrid, Spain; 4 Unidad de Neuropatología, Fundación Hospital Alcorcón, Alcorcón, Madrid, Spain; 5 Centro Nacional de Epidemiología, Instituto de Salud Carlos III, Madrid, Spain

## Abstract

**Background:**

The 14-3-3 test appears to be a valuable aid for the clinical diagnosis of sporadic Creutzfeldt-Jakob disease (sCJD) in selected populations. However, its usefulness in routine practice has been challenged. In this study, the influence of the clinical context on the performance of the 14-3-3 test for the diagnosis of sCJD is investigated through the analysis of a large prospective clinical series.

**Methods:**

Six hundred seventy-two Spanish patients with clinically suspected sCJD were analyzed. Clinical classification at sample reception according to the World Health Organization's (WHO) criteria (excluding the 14-3-3 test result) was used to explore the influence of the clinical context on the pre-test probabilities, and positive (PPV) and negative (NPV) predictive values of the 14-3-3 test.

**Results:**

Predictive values of the test varied greatly according to the initial clinical classification: PPV of 98.8%, 96.5% and 45.0%, and NPV of 26.1%, 66.6% and 100% for probable sCJDi (n = 115), possible sCJDi (n = 73) and non-sCJDi (n = 484) cases, respectively. According to multivariate and Bayesian analyses, these values represent an improvement of diagnostic certainty compared to clinical data alone.

**Conclusion:**

In three different contexts of sCJD suspicion, the 14-3-3 assay provides useful information complementary to clinical and electroencephalographic (EEG) data. The test is most useful supporting a clinical impression, whilst it may show deceptive when it is not in agreement with clinical data.

## Background

At present, the definitive diagnosis of sporadic Creutzfeldt-Jakob disease (sCJD) requires the neuropathological examination of affected brain tissues. However, several ancillary tests may add support to the clinical findings. In particular, the identification of periodic sharp wave complexes (PSWCs) in electroencephalographic (EEG) records [1] and 14-3-3 protein in cerebrospinal fluid (CSF) [2] have proved to be useful, and are included among current diagnostic criteria [3].

The use of the 14-3-3 test for the diagnosis of prion diseases was described in 1996 [2]. This protein is a reliable marker of rapid neuronal destruction [4], and has been detected in the CSF in several progressive neurological disorders [2,5-7]. The performance of the 14-3-3 test has been analyzed mainly on selected populations with predefined patients and control groups, yielding sensitivity and specificity values around 94% and 84%, respectively [8]. However, the populations studied may not reflect the real situation in clinical practice, and it is well known that the parameters of sensitivity and specificity are less useful for the clinicians than predictive values. We describe our experience in a reference laboratory dedicated to assist through molecular testing in the diagnosis of prion diseases in Spain. In this cohort of suspected sCJD cases, we have examined the performance of the 14-3-3 test and its impact on diagnostic certainty through the analysis of the predictive values in 3 different clinical contexts.

## Methods

### Patients and classification algorithm

All samples analyzed in this study were obtained from patients with suspected prion diseases, submitted to our laboratory for diagnostic purposes under the guidelines of the Spanish National Referral and Surveillance system. No healthy controls are included in the analysis. For this study, all samples were coded and personal information dissociated from the test results, according to local legislation at the time of analysis. This research does not include identifiable human material or identifiable data. The Bioethics and Animal Welfare Committee from the Instituto de Salud Carlos III (Spanish Ministry of Health) evaluated this research work and granted a positive opinion stating that the work conformed the Helsinki Declaration and local legislation (Minutes from the April 18th, 2006 meeting).

**Figure 1:**
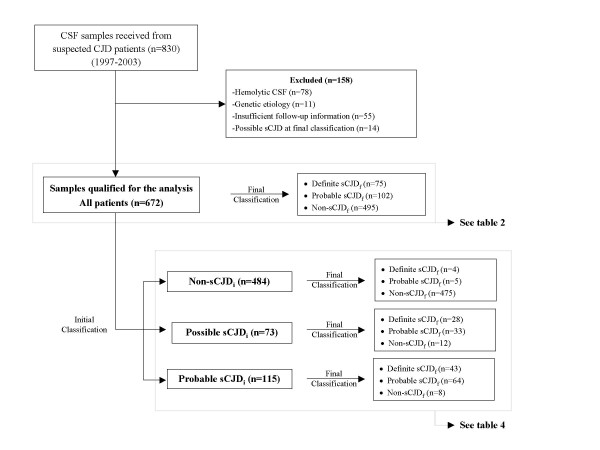
Diagnostic and classification algorithm applied to our series of clinically suspected sCJD cases.

The population under study included patients with suspected prion diseases, whose CSF samples were submitted to our laboratory from January 1997 to December 2003. Spain countrywide-patients were analyzed. Cloudy, bloodstained, or hemolytic CSF samples were excluded from the analysis (Figure 1). In order to avoid false-positive 14-3-3 results from slightly hematic/hemolytic samples (not perceptible by eye inspection), we checked the hemoglobin content of each sample by Combur strip test (Combur10 Test M, Roche). Samples yielding hemoglobin signals equivalent to 250 erythrocytes/μl or higher were not analyzed. We applied World Health Organization's criteria (WHO's criteria) (Table 1, see ref. [3]) excluding the 14-3-3 test results in order to obtain an *initial *and a *final classification *of each case; hereafter denoted by subscripts "i" or "f" at the end of the classificatory term (i.e. sCJD_i _or sCJD_f_).

For the purpose of analysis, *initial classification *was based on clinical records at the time of sample reception before the performance of the 14-3-3 test. Patients initially fulfilling WHO's criteria were classified either as possible sCJD_i _or probable sCJD_i_, while the rest of cases were classified as non-sCJD_i_.

*Final classification *of patients was accomplished by obtaining and reviewing detailed clinical, epidemiological, and neuropathological information through a structured questionnaire sent by hospital personnel, and exhaustive follow-up contact with the referring physicians and the Spanish National Human Transmissible Spongiform Encephalopathies Registry [9]. Patients finally fulfilling WHO's criteria (without 14-3-3 test results) were classified as definite sCJD_f _or probable sCJD_f_, while the remaining patients were classified as non-sCJD_f _cases. Since one of the main aims of this study was the analysis of the predictive values of the 14-3-3- test in a real practice situation, both definite and probable sCJD_f _cases at final diagnosis were considered as gold standard.

The classification algorithm applied to our population is depicted in Figure 1. During the 7-year period covered by the study, we received 830 CSF samples. Patients with hematic/hemolytic CSF samples, insufficient clinical information, genetic etiology, or possible sCJD_f _at final classification were excluded. Overall, 672 patients qualified for the study (Figure 1). The initial classification of these patients at the time of sample reception according to WHO's criteria excluding the 14-3-3 test protein included 73 possible sCJD_i_, 115 probable sCJD_i_, and 484 non-sCJD_i_. Final classification of these patients encompassed 75 definite sCJD_f _(pathologically confirmed), 102 probable sCJD_f_, and 495 non-sCJD_f _cases (patients pathologically excluded, showing a clinical course not compatible with prion disease or with alternative diagnoses).

### Analysis of the polymorphism at codon 129 of *PRNP gene*

Genomic DNA was directly extracted from peripheral blood with DNAzol Genomic DNA Isolation Reagent (Molecular Research Center, Inc.), according to the manufacturer's instructions. The coding region of *PRNP *gene was amplified by PCR. The polymorphism at codon 129 was examined either by restriction enzyme digestion with *Nsp*I or by DNA sequencing of the amplicons.

### Demographic and clinical data

**Figure 2:**
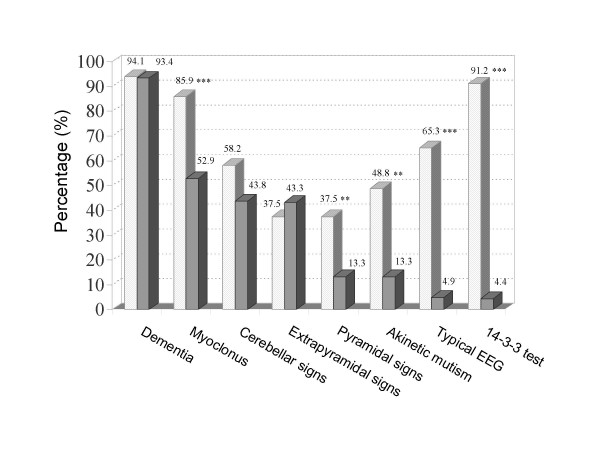
**Frequency of clinical and paraclinical data in sCJDf (light shaded bars) and non-sCJDf patients (dark shaded bars)**. * p < 0.05, ** p < 0.01, and *** p < 0.001. sCJD_f _includes both probable and definite patients.

In this series of 672 patients, sCJD_f _(definite and probable) and non-sCJD_f _cases had similar gender distribution and age at onset: sCJD_f _(94/177 females, median age 70.4 y, SD 8.4 y, range 45.1–86.9 y) vs. non-sCJD_f _(232/495 females, median age 69.1 y, SD 12.9, range 27.9–81.7 y). The three most frequent symptoms among sCJD_f _cases were dementia, myoclonus and cerebellar signs (Figure 2). The distribution of the polymorphisms at codon 129 was 69.5% 129MM, 16.1% 129MV, and 14.4% 129VV for sCJD_f _(n = 118), and 43.6% 129MM, 44.3% 129MV, and 12.1% 129VV for non-sCJD_f _(n = 289).

### 14-3-3 test

The presence of 14-3-3 protein in CSF was analyzed by the method previously described [2] with minor modifications [10]. Briefly, proteins in 20 μl CSF were mixed with 15 μl of Laemmli buffer (2x) containing 0.1 M dithiothreitol (Bio-Rad) and solubilized at 100°C for 5 minutes. Proteins were then separated by electrophoresis in sodium dodecyl sulfate-polyacrylamide gels (4% stacking, 12% separating) and transferred to nitrocellulose membranes (Amersham, Biosciences). Immunostaining was performed after membrane blocking (Super Block, Pierce) by overnight incubation at room temperature with an anti 14-3-3β polyclonal rabbit antibody (sc-629, Santa Cruz Biotecnology). The 14-3-3 antigens were detected with alkaline phosphatase conjugated to goat anti-rabbit IgG and developed by colorimetric reaction with NBT/BCIP (Pierce Biotechnology, Inc). The immunoreactivity of each sample at the 30 kDa. band was assessed by two different observers blinded to the clinical data, in at least two different experiments. According to the presence or absence of immunoreactivity at the 30 kDa. band, samples were classified as negative or positive. As the test is based on a semiquantitative technique, some samples could not be clearly classified as negative or positive compared to reference standards that included a positive control from a sCJD patient and a weakly positive control sample from a non-CJD patient. For the purpose of this study and the diagnostic service provided to clinicians, trace samples with immunoreactivity lower than the weakly positive control sample were considered negative (see discussion).

### Statistical analysis

Univariate comparisons between groups were performed with the χ^2 ^and Mann-Whitney tests, as appropriate. Multivariate logistic regression analysis was performed taking the final diagnosis as dependent variable (sCJD_f _/non-sCJD_f_) and the clinical, EEG and 14-3-3 protein data as covariates. Bayesian analysis was performed calculating the positive (LR+) and negative (LR-) likelihood ratio statistics. The multivariate and Bayesian analyses were limited to definite sCJD_f _and non-sCJD_f_. All the statistical calculations were performed with SPSS software.

## Results

### Performance of the 14-3-3 test

The prevalence of sCJD_f _(definite and probable) in the overall cohort of suspected cases (n = 672) was 26.3%. The performance parameters of the 14-3-3 test are shown in Table 2. The efficiency of the test, i.e. the proportion of subjects correctly classified, was 94.5%. Among sCJD_f _patients, no statistically significant differences were found between 14-3-3-positive and 14-3-3-negative cases for any of the parameters studied (Table 3). However, a tendency to a later onset and longer duration of disease was observed among 14-3-3-negative sCJD_f _patients, as previously described [11], and in agreement with the higher sensitivity observed in classic sCJD cases compared with non-classical presentations [12].

### Influence of the clinical context on the 14-3-3 test

Table 4 summarizes the performance of the test in three categories of patients classified upon sample reception according to WHO's criteria excluding 14-3-3 test results.

The prevalence of sCJD_f _within the probable sCJD_i _subgroup (n = 115) was 93.0%. Ninety sCJD_f _were 14-3-3-positive, and the remaining 17 cases were false-negatives (7 definite and 10 probable sCJD_f _cases). Two false-positive cases met diagnostic criteria for probable sCJD_i_, but were finally diagnosed as vascular dementia. These two false-positive patients showed rapidly progressive dementia, myoclonus, visual disturbances, and PSWCs. Within this group, 6 patients initially fulfilled sCJD criteria and displayed PSWCs, but finally were classified as non-sCJD cases. Four of these patients were confirmed as alternative diagnoses by post-mortem examination and included 2 patients with a neurological paraneoplastic syndrome, one patient with Lewy body dementia, and one patient with toxic encephalopathy; the remaining two patients after prolonged course of the disease did not meet criteria for CJD and were classified by the referring clinicians as probable Alzheimer's disease and Lewy body dementia (disease duration > 3 years), respectively. In the category of possible sCJD_i _(n = 73 cases), the prevalence of sCJD_f _was 83.6%. Twelve patients were later reclassified as non-sCJD_f_. There were two false-positive cases: one with definite Lewy Body dementia, and one with vascular dementia. Five 14-3-3-negative patients were finally classified as sCJD_f_. Within this group, 5 patients gave false negative results, and included one patient confirmed as sCJD at post-mortem examination and 4 patients that showed PSWCs later along the course of their disease.

Within the category of non-sCJD_i _(n = 484), the prevalence of sCJD_f _was 1.9%. All cases classified as definite sCJD_f _(n = 4) or probable sCJD_f _(n = 5) had a 14-3-3-positive test. The four definite sCJD_f _cases never showed PSWCs, and two of them did not display dementia at onset. The other five probable sCJD_f _cases developed dementia and their EEG became typical at a later stage of disease once the 14-3-3 analysis had been performed. The 14-3-3 test also yielded 11 false-positive results that never fulfilled clinical criteria for sCJD. Interestingly, there were no false-negative results when the initial diagnosis was non-sCJD_i_.

### Improving diagnostic accuracy

Univariate analyses comparing patients with final diagnoses of probable or definite sCJD_f _versus non-sCJD_f _cases showed, as expected, that myoclonus, pyramidal signs, akinetic mutism, as well as PSWCs, 14-3-3 positive result, and homozygosis at codon 129, were all associated with sCJD. In order to explore whether the 14-3-3 test provide information of diagnostic relevance when added to WHO's criteria, we applied multivariate analysis and Bayesian statistics. For definite cases, multivariate analysis including the clinical, and the EEG or the 14-3-3 data in two separate models, showed that the only predictive factors for sCJD were myoclonus (OR: 9.4; 95% CI: 1.2–70.9), akinetic mutism (OR: 16.4; 95% CI: 1.5–202.5), PSWCs (OR: 5.8; 95% CI: 1.5–21.9), and positive 14-3-3 test (OR: 146.5; 95% CI: 15.1–1415.6).

According to Bayesian analysis, the LR+ for WHO's criteria alone was 23.4 (95 % CI: 15.2–36.1) and LR- was 0.06 (95 % CI: 0.02–0.14). The LR+ for the 14-3-3 test alone was 29.5 (95 % CI: 17.8–48.8) and the LR- was 0.11 (95 % CI: 0.06–0.21). The LR+ and LR- for combined WHO's criteria and 14-3-3 test were 103.9 (95 % CI: 39.0–277.2) and 0.2 (95 % CI: 0.1–0.3), respectively. These results indicate that the inclusion of the 14-3-3 test produces a moderate but significant increase in the LR+ compared to WHO's criteria alone. In other words, given a pre-test probability of disease in our cohort of 13.2% (prevalence of sCJD_f _restricted to definite cases), after applying WHO's criteria alone, the PPV among patients that fulfilled criteria was 78.0%, while the NPV was 99.2%. Considering the 14-3-3 test alone, the PPV was 81.7%, while the NPV was 98.4%. When both analyses were combined as serial tests, the PPV for patients that fulfilled criteria and had a 14-3-3 result was 94.0%, while the NPV for patients that neither fulfilled criteria nor had a positive 14-3-3 result was 100%.

## Discussion

Since the introduction of the 14-3-3 test for the diagnosis of sCJD [2], the test has been shown to be feasible, reliable and valid under controlled conditions, and it is therefore now routine practice in the diagnostic workup of suspected CJD patients. According to published results, the sensitivity and specificity of the test are about 93% and 84%, respectively [8]. PPV and NPV are highly dependent on the population tested, and are consequently relevant only when applied within similar clinical contexts. Moreover, the importance of the "context of suspected sCJD" has been emphasized but not defined [8]. In this study, we have followed prospectively a series of 672 patients with a clinical suspicion of sCJD, whose samples were submitted for evaluation to our laboratory according to the guidelines of the Spanish National Health System. The analysis of this series provides an operational frame for classification and definition of such context of suspected sCJD. This context, linked to the interpretation of 14-3-3 test results, has not yet been empirically illustrated in large series of patients. Unfortunately, the large EUROCJD (European and Allied Countries Collaborative Study Group of CJD) research database, generated mainly for surveillance, is useful for evaluation of sensitivity and specificity, but it does not contain information on controls free from human transmissible spongiform encephalopathies.

The use of three subgroups of patients classified according to WHO's criteria at sample reception is operationally feasible; although it requires access to EEG results for 14-3-3 test interpretation. Our own results suggest that a positive 14-3-3 test in a patient with possible or probable sCJD_i _at initial classification is nearly always a true positive, whilst a negative test in a low-risk individual is almost certainly a true negative. In contrast, a negative result in a patient initially classified as possible or probable sCJD_i _should not rule out the diagnosis, and a positive test in a low-risk patient could be a false positive. In summary, the 14-3-3 assay is most useful supporting a clinical suspicion, but it may show deceptive when it is not in agreement with clinical data. In the three subgroups the PPV lay above their respective sCJD prevalence, indicating that the test provided useful information complementary to the clinical and EEG data. This observation was further supported by multivariate and Bayesian analyses.

Recently, Huang and collaborators [13] investigated the performance of the 14-3-3 test in a series of patients with rapidly progressive dementia. They concluded that the 14-3-3 test might not differentiate CJD from other rapidly progressive dementias. Similar results were previously found by Burkhard and collaborators [6] in patients with unselected dementia, since the PPV for this group was low (28%, 95% CI: 7%–40%). Geschwind and collaborators [14] also challenged the utility of the 14-3-3 test after finding a modest sensitivity in their retrospective analysis of 32 definite sCJD cases. In the same line, more recently, Blennow and collaborators have reported low sensitivity (44%) and PPV (36%) values for a small cohort (n = 36) of clinically suspected CJD [15], suggesting that methodological differences are responsible for the diverse results described in the literature. The risks of the indiscriminate use of the 14-3-3 test have been also pointed out by other authors [6]. Our results on the interpretation of the test shed light on these discrepancies and indicate that clinical profile should be thoroughly evaluated at sample reception, when the test would be most useful in clinical decision-making.

Analysis of our series indicated slightly lower sensitivity and higher specificity values than those previously described [2,11]. In our opinion, these differences concern mainly the interpretation of trace results, and the presence of small amounts of blood contaminating the CSF samples. The 14-3-3 determination in CSF by immunoblotting implies some difficulties for the interpretation of trace results, i.e. those that are neither clearly negative nor positive. We have interpreted trace results as negative. This practice avoids the exclusion of other potentially treatable diseases, since a 14-3-3 positive result might be interpreted by the clinician as a confirmation of the clinical suspicion of this so far untreatable disease. On the other hand, this strategy increases the number of false-negative results that according to other authors pose an important risk due to the transmissibility of the disease and the potential hazards derived from patient care and sample manipulation [14]. However, as we have presently discussed, a negative result in a patient with high clinical suspicion does not exclude the diagnosis of sCJD. Furthermore, our strategy allows optimizing the PPV of the test.

The second reason for discrepancy might be related to blood contamination of samples. In our hands, a small percentage of samples were contaminated by blood at levels undetectable by eye inspection, but sufficient to produce false-positives due to the presence of serum proteins in the CSF (data not shown). Accordingly, samples yielding hemoglobin levels equivalent to more than 250 erythrocytes/μl were excluded. The inclusion of this pre-test analysis allowed us to increase the specificity of the 14-3-3 test by rejecting potential false-positive samples.

Our study has several features of clinical relevance. Firstly, it is a prospective analysis of all sCJD suspected patients, and accordingly is not circumscribed to a subset of definite and control cases. Therefore, it represents a real practice situation and allows the estimation of predictive values of relevance for clinical decision-making. Secondly, it includes a large sample of patients attended by many physicians at a variety of institutions, which increases the external validity of the study. Thirdly, the period of observation spanned 7 years, long enough to obtain full follow-up information from most sCJD cases. Finally, the 14-3-3 test was performed and interpreted by a limited team using standardized techniques, which reduces the inter-rater variability of the test. Unfortunately, our study has also several limitations. Most important, we still have a low rate of autopsies of 41.5%, and we do not know the PrP^Sc ^isoform in a sufficient number of cases to be included in this study. Moreover, we also had a significant number of losses from insufficient follow-up. These cases always raise concerns about their possible peculiarities (e.g. long duration 129VV homozygotes) [16], and the consequent biases derived from their exclusion.

The implementation of quantitative protocols for the determination of 14-3-3 protein in CSF [17,18], together with the determination of other biological markers and EEG and MRI data, all of them considered in the particular clinical context of each patient, might result in the near future into the development of protocols with high predictive values for the *in vivo *diagnosis of sCJD.

## Conclusion

In conclusion, this work helps to define the concept of clinical context for the correct use and interpretation of the 14-3-3 test. Our results demonstrate that the combination of clinical classification at the time of test performance together with the results of the 14-3-3 test provides physicians with a valuable diagnostic aid.

## Abbreviations

LR+: positive likelihood ratio; LR-: negative likelihood ratio; NPV: negative predictive value; OR: Odds ratio; PPV: positive predictive value; PSWCs: periodic sharp wave complexes; sCJD: sporadic Creutzfeldt-Jakob disease

## Competing interests

The author(s) declare that they have no competing interests.

## Authors' contributions

NCC, AJH, and MC drafted the manuscript, conceived and designed the study concept, and coordinated the study. CA, LV, RH and LC performed the immunoassays and molecular tests, MSM participated in the statistical analysis, AR performed the post-mortem examinations. AR and JPC aided in the preparation of the manuscript and provided critical analysis of the manuscript.

All authors read and approved the final manuscript.

## Pre-publication history

The pre-publication history for this paper can be accessed here:



## Figures and Tables

**Table 1:** World Health Organization's criteria for the classification of sCJD

I.	Rapidly progressive dementia
II.	A. Myoclonus B. Visual or Cerebellar problems C. Pyramidal or extrapyramidal features D. Akinetic mutism
III.	A. Typical EEG B. Positive 14-3-3

Possible sCJD: I and 2 of II and duration less than 2 years

Probable sCJD: I and 2 of II and IIIA or possible CJD and IIIB

Definite sCJD: Neuropathologically/immunocytochemically confirmed

**Table 2:** Contingency table and performance values of the 14-3-3 test in a population of clinically suspected sCJD cases

		***All cases, *n = 672**	
*Final diagnosis*		***sCJD***_*f*_	***non-sCJD***_*f*_

*14-3-3 result*	**Pos.**	155 [67]	15
	
	**Neg.**	22 [8]	480

*Performance values (%)*			
**PPV**		91.2 (85.6–94.8)	
**NPV**		95.6 (93.3–97.2)	
**Sensitivity**		87.6 (81.6–91.9)	
**Specificity**		96.9 (94.9–98.2)	
**Efficiency**		94.5 (92.5–96.0)	
**Prevalence**		26.3 (23.2–29.8)	

*sCJDf *includes both probable (WHO's criteria without the inclusion of the 14-3-3 test results) and definite cases at final diagnosis. Definite cases are shown in square brackets. Confidence intervals at 95% level are shown in parentheses.

**Table 3:** Age at onset, survival time, lumbar puncture (LP) timing, presence of PSWCs in EEG, and genotype at codon 129 of *PRNP *gene of 14-3-3-positive and 14-3-3-negative sCJD_**f **_patients

*14-3-3 result*	**Age at onset, years**	**Survival time, months**	**Time from onset to LP, months**	**Time from LP to death, months**	**PSWCs in EEG, %**	**M129V, %**
**Positive**	69.8 (45.1–86.9), n= 144	4.7 (0.5–48.8), n= 138	2.6 (0.7–27.2), n= 125	1.2 (0–30.8), n= 119	83.9%, n= 112	68.0%MM, 15.5%MV, 16.5%VV, n= 103
**Negative**	73.4 (59.1–82.3), n= 21	5.4 (0.4–29.9), n= 15	2.9 (0.2–13.9), n= 17	1.3 (0–26.8), n= 14	81.8%, n= 22	80.0%MM, 20.0%MV, 0%VV, n= 15

*sCJD*_*f *_includes both probable and definite cases. The data of age, survival time, and LP timing correspond to median and range values.

**Table 4:** Contingency tables and performance of the 14-3-3 test according to the initial clinical classification

		*Initial diagnosis*					
		
		***Probable CJD,***_***i ***_**n= 115**		***Possible CJD,***_***i ***_**n= 73**		***non-sCJD,***_***i ***_**n= 484**	
*Final diagnosis*		***sCJD***_***f***_	***non-sCJD***_***f***_	***sCJD***_***f***_	***non-sCJD***_***f***_	***sCJD***_***f***_	***non-sCJD***_***f***_

*14-3-3 result*	**Pos.**	90 [36]	2	56 [27]	2	9 [4]	11
	
	**Neg.**	17 [7]	6	5 [1]	10	0	464

*Performance values (%)*							
**PPV**		97.8 (91.6–99.6)		96.5 (87.0–99.4)		45.0 (23.8–68.0)	
**NPV**		26.1 (11.1–48.7)		66.6 (38.7–87.0)		100 (99.0–100.0)	
**Sensitivity**		84.1 (75.5–90.2)		91.8 (81.2–96.9)		100 (62.9–100.0)	
**Specificity**		75.0 (35.5–95.5)		83.3 (50.9–97.1)		97.7 (95.8–98.8)	
**Efficiency**		83.5 (75.6–89.3)		90.4 (81.3–95.6)		97.7 (95.9–98.8)	
**Prevalence**		93.0 (86.7–96.7)		83.6 (73.3–90.5)		1.9 (0.9–3.6)	

*sCJDf *includes both probable (WHO's criteria without the inclusion of the 14-3-3 test results) and definite cases at final diagnosis. Definite cases are shown in square brackets. Confidence intervals at 95% level are shown in parentheses.
